# Expanding *SPTAN1* monoallelic variant associated disorders: From epileptic encephalopathy to pure spastic paraplegia and ataxia

**DOI:** 10.1016/j.gim.2022.09.013

**Published:** 2023-01

**Authors:** Heba Morsy, Mehdi Benkirane, Elisa Cali, Clarissa Rocca, Kristina Zhelcheska, Valentina Cipriani, Evangelia Galanaki, Reza Maroofian, Stephanie Efthymiou, David Murphy, Mary O’Driscoll, Mohnish Suri, Siddharth Banka, Jill Clayton-Smith, Thomas Wright, Melody Redman, Jennifer A. Bassetti, Mathilde Nizon, Benjamin Cogne, Rami Abu Jamra, Tobias Bartolomaeus, Marion Heruth, Ilona Krey, Janina Gburek-Augustat, Dagmar Wieczorek, Felix Gattermann, Meriel Mcentagart, Alice Goldenberg, Lucie Guyant-Marechal, Hector Garcia-Moreno, Paola Giunti, Brigitte Chabrol, Severine Bacrot, Roger Buissonnière, Virginie Magry, Vykuntaraju K. Gowda, Varunvenkat M. Srinivasan, Béla Melegh, András Szabó, Katalin Sümegi, Mireille Cossée, Monica Ziff, Russell Butterfield, David Hunt, Georgina Bird-Lieberman, Michael Hanna, Michel Koenig, Michael Stankewich, Jana Vandrovcova, Henry Houlden, Genomics England Research Consortium, J.C. Ambrose, P. Arumugam, E.L. Baple, M. Bleda, F. Boardman-Pretty, J.M. Boissiere, C.R. Boustred, H. Brittain, M.J. Caulfield, G.C. Chan, C.E.H. Craig, L.C. Daugherty, A. de Burca, A. Devereau, G. Elgar, R.E. Foulger, T. Fowler, P. Furió-Tarí, J.M. Hackett, D. Halai, A. Hamblin, S. Henderson, J.E. Holman, T.J.P. Hubbard, K. Ibáñez, R. Jackson, L.J. Jones, D. Kasperaviciute, M. Kayikci, L. Lahnstein, K. Lawson, S.E.A. Leigh, I.U.S. Leong, F.J. Lopez, F. Maleady-Crowe, J. Mason, E.M. McDonagh, L. Moutsianas, M. Mueller, N. Murugaesu, A.C. Need, C.A. Odhams, C. Patch, D. Perez-Gil, D. Polychronopoulos, J. Pullinger, T. Rahim, A. Rendon, P. Riesgo-Ferreiro, T. Rogers, M. Ryten, K. Savage, K. Sawant, R.H. Scott, A. Siddiq, A. Sieghart, D. Smedley, K.R. Smith, A. Sosinsky, W. Spooner, H.E. Stevens, A. Stuckey, R. Sultana, E.R.A. Thomas, S.R. Thompson, C. Tregidgo, A. Tucci, E. Walsh, S.A. Watters, M.J. Welland, E. Williams, K. Witkowska, S.M. Wood, M. Zarowiecki

**Affiliations:** 1 Department of Neuromuscular Disorders, UCL Institute of Neurology, University College London, Queen Square, London, United Kingdom; 2 Department of Human Genetics, Medical Research Institute, Alexandria University, Alexandria, Egypt; 3 Department of Molecular Genetic, University Institute for Clinical Research, Montpellier University Hospital, PhyMedExp, CNRS UMR 9214, INSERM U1046, Montpellier, France; 4 William Harvey Research Institute, Barts & The London School of Medicine and Dentistry, Queen Mary University of London, London, United Kingdom; 5 UCL Institute of Ophthalmology, University College London, London, United Kingdom; 6 Department of Clinical and Movement Neurosciences, UCL Queen Square Institute of Neurology, University College London, London, United Kingdom; 7 West Midlands Regional Clinical Genetics Service, Birmingham Health Partners, Birmingham Women’s and Children’s Hospital NHS Foundation Trust, Birmingham, United Kingdom; 8 Nottingham Clinical Genetics Service, Nottingham University Hospitals NHS Trust, Nottingham, United Kingdom; 9 Division of Evolution, Infection and Genomics, School of Biological Sciences, Faculty of Biology, Medicine and Health, University of Manchester, Manchester, United Kingdom; 10 Manchester Centre for Genomic Medicine, Manchester University NHS Foundation Trust, Manchester, United Kingdom; 11 Department of Clinical Genetics, Chapel Allerton Hospital, Leeds Teaching Hospitals NHS Trust, Leeds, United Kingdom; 12 Department of Pediatrics, Weill Cornell Medicine, New York, NY; 13 Thorax Institute, Nantes University, CNRS, INSERM, Nantes, France; 14 Department of Medical Genetics, Nantes University Hospital, Nantes, France; 15 MVZ for Diagnostic and Therapy, Leipziger Land, Leipzig, Germany; 16 Institute of Human Genetics, University of Leipzig Medical Center, University of Leipzig, Leipzig, Germany; 17 Division of Neuropediatrics, Hospital for Children and Adolescents, University Hospital Leipzig, Leipzig, Germany; 18 Institute of Human Genetics, Medical Faculty, University Hospital Düsseldorf, Düsseldorf, Germany; 19 Medical Genetics, St George's University Hospitals NHS Foundation Trust, London, United Kingdom; 20 Department of Medical Genetics, Rouen University Hospital, Rouen, France; 21 Department of Pediatric Neurology, Marseille University Hospital, Marseille, France; 22 Department of Neurogenetics, National Hospital for Neurology and Neurosurgery, University College London Hospitals NHS Foundation Trust, London, United Kingdom; 23 Ataxia Centre, Department of Clinical and Movement Neurosciences, UCL Queen Square Institute of Neurology, London, United Kingdom; 24 Reference Center for Inherited Metabolic Diseases, Marseille University Hospital, Marseille, France; 25 Department of Molecular Genetics, Versailles Hospital, Versailles, France; 26 Department of Pediatrics, Versailles Hospital, Versailles, France; 27 Department of Molecular Genetics, Amiens-Picardie University Hospital, Amiens, France; 28 Department of Pediatric Neurology, Indira Gandhi Institute of Child Health, Bangalore, India; 29 Department of Medical Genetics, Clinical Centre, School of Medicine, University of Pécs, Pécs, Hungary; 30 Department of Biochemistry and Medical Chemistry, Medical School, University of Pécs, Pécs, Hungary; 31 Clinical Genetics Department, Great Ormond Street Hospital for Children NHS Foundation Trust, London, United Kingdom; 32 Division of Pediatric Neurology, Department of Pediatrics, University of Utah School of Medicine, University of Utah Health, Salt Lake City, UT; 33 Wessex Clinical Genetics Service, Princess Anne Hospital, Southampton, United Kigngdom; 34 Southampton Children’s Hospital, University Hospital Southampton NHS Foundation Trust, Southampton, United Kingdom; 35 Department of Pathology, Yale School of Medicine, New Haven, CT

**Keywords:** Developmental delay, Developmental epileptic encephalopathy, Hereditary ataxia, Hereditary spastic paraplegia, SPTAN1

## Abstract

**Purpose:**

Nonerythrocytic αII-spectrin (*SPTAN1*) variants have been previously associated with intellectual disability and epilepsy. We conducted this study to delineate the phenotypic spectrum of *SPTAN1* variants.

**Methods:**

We carried out *SPTAN1* gene enrichment analysis in the rare disease component of the 100,000 Genomes Project and screened 100,000 Genomes Project, DECIPHER database, and GeneMatcher to identify individuals with *SPTAN1* variants. Functional studies were performed on fibroblasts from 2 patients.

**Results:**

Statistically significant enrichment of rare (minor allele frequency < 1 × 10^–5^) probably damaging *SPTAN1* variants was identified in families with hereditary ataxia (HA) or hereditary spastic paraplegia (HSP) (12/1142 cases vs 52/23,847 controls, p = 2.8 × 10^–5^). We identified 31 individuals carrying *SPTAN1* heterozygous variants or deletions. A total of 10 patients presented with pure or complex HSP/HA. The remaining 21 patients had developmental delay and seizures. Irregular αII-spectrin aggregation was noted in fibroblasts derived from 2 patients with p.(Arg19Trp) and p.(Glu2207del) variants.

**Conclusion:**

We found that *SPTAN1* is a genetic cause of neurodevelopmental disorder, which we classified into 3 distinct subgroups. The first comprises developmental epileptic encephalopathy. The second group exhibits milder phenotypes of developmental delay with or without seizures. The final group accounts for patients with pure or complex HSP/HA.

## Introduction

The αII-spectrin gene, *SPTAN1* (OMIM 182810), encodes a membrane scaffolding protein that plays an important role in the maintenance of integrity of myelinated axons, axonal development, and synaptogenesis.^1^ Heterozygous *SPTAN1* pathogenic variants have been previously reported with variable phenotypes, most frequently causing mild to severe developmental epileptic encephalopathy (DEE) and developmental delay (DD)^2^ and rarely with hereditary motor neuropathy and autosomal recessive hereditary spastic paraplegia (HSP).^3^^,^^4^ A mouse model harboring αII-spectrin missense variant (p.Arg1098Gln) was reported to develop progressive ataxia with global neurodegeneration and seizures.^5^ On the basis of these findings, we carried out a *SPTAN1* gene enrichment analysis in the 100,000 Genomes Project (100K GP)^6^ and identified a statistically significant enrichment for rare probably damaging variants in hereditary ataxia (HA) and HSP groups. In this study, we present an extended phenotypic spectrum of neurologic syndromes caused by pathogenic variations of *SPTAN1* gene*.*

## Materials and Methods

### Patients

Our initial cohort comprised 100K GP neurology patients.^6^ All 100K GP genomes were previously screened for single nucleotide variants, small insertions/deletions, structural variants (SVs) or copy number variants (CNVs), and short tandem repeats in relevant genes from the PanelApp virtual gene panels (Genomics England).^7^ We then screened DECIPHER database cohort for patients carrying single nucleotide variants and/or SVs/CNVs in *SPTAN1* gene.^8^ Additional families were subsequently recruited through GeneMatcher.^9^ All coding variants reported in this article are with reference to *SPTAN1* RefSeq: NM_001130438.3 transcript. All procedures adhered to the principles set out in the Declaration of Helsinki and all patients/their guardians included in the study consented to participation according to ethical approval of the recruiting center.

### Gene enrichment analysis

Case-control gene enrichment analysis was performed within the rare disease component of the 100K GP. Cases were defined as all 100K GP probands recruited under HA/HSP, whereas controls were all remaining probands recruited into the 100K GP except for those with neurologic and neurodevelopmental disorders or metabolic disorders. Enrichment of *SPTAN1* rare, probably damaging variants in cases compared with controls was assessed via a two-sided Fisher exact test. The contributing variants were defined as rare (minor allele frequency < 1 × 10^–5^) and either protein-truncating variants or missense variants predicted to be pathogenic by 2 in silico tools (Combined Annotation Dependent Depletion [CADD]^10^ and Polymorphism Phenotyping [PolyPhen]^11^).

### Functional studies

Fibroblasts derived from patient 1 (p. Arg19Trp) and patient 29 (p. Glu2207del) were used to test the functional effects of *SPTAN1* variants on protein expression compared with that of healthy unrelated controls. Western blot analysis, immunocytochemistry, and confocal microscopy were performed as previously described.^3^

### Structural modeling of *SPTAN1* missense variants

Three-dimensional protein modeling was used to evaluate the effect of reported *SPTAN1* missense variants. Although the crystal structure for full-length αII-spectrin is unknown, crystal structures of the N-terminal tetramerization site and 2 spectrin repeat unit of chicken brain αII-spectrin have been solved (Protein Data Bank: 3F31 and 3Fb2).^12^^,^^13^ We used the Protein Homology/analogY Recognition Engine V 2.0 (Phyre2) predicted models for C-terminal and spectrin repeats 13 to 20 of αII-spectrin protein.^14^ DynaMut software was used to predict variant effect.^15^ For simulating amino acid substitutions and visualization, UCSF Chimera built-in tools were used.^16^ In addition, in silico pathogenicity prediction analysis of all missense variants identified in the study and those previously reported in literature was conducted.

## Results

### *SPTAN1* heterozygous damaging variants are enriched in families with HSP or HA

*SPTAN1* was investigated as a candidate gene for HA or HSP using gene enrichment analysis in the rare disease component of the 100K GP, which has a total of 35,422 rare disease families, including 1142 HA/HSP probands as cases and 23,847 non-neurologic/non-metabolic unrelated individuals as controls. A case-control analysis revealed a statistically significant enrichment of rare probably damaging heterozygous variants of *SPTAN1* in probands with HA or HSP (12/1142 cases vs 52/23,847 controls, p = .00002846, odds ratio = 4.8594, 95% CI = 2.5867-9.1290) (Supplemental Table 1). None of the *SPTAN1* variants found in controls were protein-truncating variants and none overlapped with any of the missense variants described in the study.

**Figure 1:**
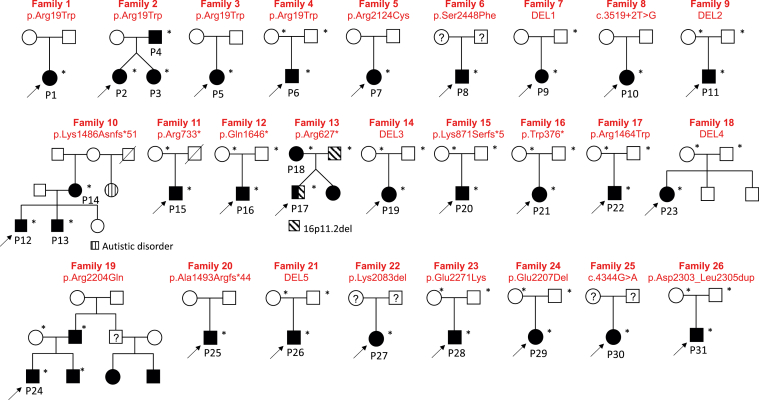
**Pedigrees of reported families with *SPTAN1* variants showing disease segregation**.

**Figure 2:**
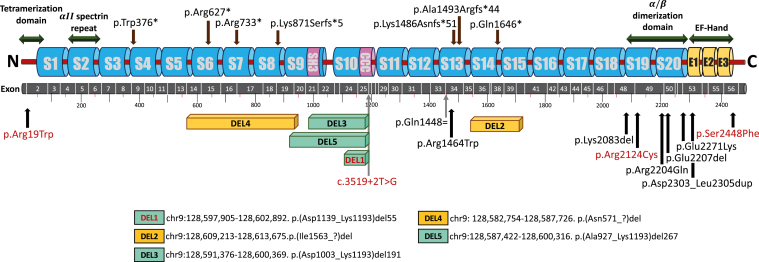
**Schematic structure of *SPTAN1* gene and its coding protein highlighting variants identified in this study.** Coding exon numbers (NM_001130438.3) are reported on the gray bar. Truncating variants are indicated on the top. Missenses, in-frame deletion/insertion, and splice variants are on the bottom. Deletion 1, 3, and 5 (green) remain in frame, whereas predictions for deletions 2 and 4 (orange) are not available. p.Gln1448= (c.4344G>A) is predicted to affect exon 33 donor splice site, based on maxENTScan (predicting splice sites using ‘Maximum Entropy Principle’) (maxENT score wild-type 6.99 → 3.84 mutant). The splice altering variant (NC_000009.12(*SPTAN1*_v001):c.3519+2T>G) predicted to alter exon 25 canonical donor splice site (maxENT score wild-type 10.28 → 2.63 mutant). Variants identified in patients presenting with HSP/HA are highlighted in red. All other variants are represented in black.

Subsequently, we screened 100K GP neurology cohort (16,014 individuals with neurodevelopmental disorders) for probably damaging *SPTAN1* variants in families with spasticity and ataxia in addition to the previously described phenotypes of seizures and/or intellectual disability (ID). We identified 11 patients from 9 families (Table 1, Figures 1 and 2). Patients 1 to 4 had pure HSP phenotype and shared the same *SPTAN1* variant, p.(Arg19Trp). Later-onset and a more complex phenotype was noted in patient 8 who harbored p.(Ser2448Phe) variant. Although patient 7 was recruited under early-onset dystonia phenotype, she presented with abnormal eye movements, ataxia, myoclonus, and dyspraxia and had *SPTAN1* variant, p.(Arg2124Cys). Patient 10, who had pure ataxia, harbored a heterozygous splice alteration in *SPTAN1* (NC_000009.12[*SPTAN1*_v001]:c.3519+2T>G). *SPTAN1* gene was also screened for CNVs/SVs in the 100K GP and 1 deletion was identified. Patient 9 carried a large heterozygous in-frame deletion (DEL1), encompassing exons 25 to 27 (Supplemental Figure 1) and presented with pure HA. Both patients 9 and 10 carried sporadic *SPTAN1* variants because the de novo nature could not be confirmed owing to the unavailability of family members. Additional 3 probands with *SPTAN1* variants presenting with seizures, ID, and ataxia/spasticity were identified.

**Table 1:** Clinical and genetic findings of 31 patients reported in this study

General Information	Family 1	Family 2			Family 3	Family 4	Family 5	Family 6	Family 7	Family 8
	Patient 1	Patient 2	Patient 3	Patient 4	Patient 5	Patient 6	Patient 7	Patient 8	Patient 9	Patient 10
Sex/ethnicity	F/Asia	F/Europe	F/Europe	M/Europe	F/Europe	M/Europe	F/NA	M/Asia	F/NA	M/Europe
Age at last examination, y	40	25	25	50	17	15	32	50	72	54
Genetic findings										
c.DNA	c.55C>T	c.55C>T			c.55C>T	c.55C>T	c.6370C>T	c.7343C>T	arr{hg38}9q34.11(128,597,905-128,602,892)x1	NC_000009.12(*SPTAN1*_v001):c.3519+2T>G exon 25
Protein	p.Arg19Trp	p.Arg19Trp			p.Arg19Trp	p.Arg19Trp	p.Arg2124Cys	p.Ser2448Phe	p.(Asp1139_Lys1193)del (DEL 1)	p.?
Inheritance	Sporadic	AD			Sporadic	De novo	Sporadic	Unknown	Sporadic	Sporadic
**Phenotypic category**	**Pure HSP/ataxia**	**Pure HSP/ataxia**			**Complex HSP**	**Pure HSP**	**Pure HSP/ataxia**	**Complex HSP**	**Pure HA**	**Pure HA**
Initial symptoms	Spastic gait	Spastic gait	Spastic gait	Spastic gait	Learning disability/ spastic ataxia	Spastic gait	Spastic gait	Spastic gait	Ataxia	Ataxia
Age of onset, y	8	NA	NA	NA	10	8	NA	NA	36	35
SZ (age of onset)	–	–	–	–	Generalized (10 y)	–	–	–	–	–
Response to therapy	/	/	/	/	VAL; controlled	/	/	/	/	/
EEG	NA	NA	NA	NA	Normal	NA	NA	NA	NA	NA
Developmental history										
ID	–	–	–	–	–	–	–	+	–	–
Learning disability	–	–	–	–	+	–	–	+	–	–
Motor delay	–	–	–	–	–	–	–	–	–	–
Language delay	–	–	–	–	–	–	–	–	–	–
Microcephaly	–	–	–	–	–	–	–	–	–	–
Neurologic findings										
Ataxia	+	+	+	+	+	–	+	–	+	+
Spasticity	+	+	+	+	+	+	+	+	–	–
Extensor planter reflex	+				+	–	–	–	–	–
Abnormal eye movement	–	+	+	+	Nystagmus	–	+	Nystagmus	–	Nystagmus, esotropia
UL weakness	+	+	+	+		–	–	–	–	–
LL weakness	+	+	+	+	+	+	–	–	–	–
LL hyperreflexia	+	+	+	+	+	+	–	–	–	–
Ankle clonus					+	+	+	–	–	–
Bladder dysfunction	+	+	+	+		–	–	+	–	–
Amyotrophy	–	+	+	+		–	–	–	–	–
Myoclonus	–	–	–	–	–	–	+	+	–	–
Brain MRI	Normal	NA	NA	NA	Subcortical white mater hyper intensities	Normal	NA	Cerebellar atrophy	NA	Cerebellar atrophy marked in vermis
Other clinical features	–	Impaired vibratory sensation	–	–	–	Mild hearing loss, atrophic left kidney	Head tremors, dyspraxia, postural tremors	Fasciculations, impaired proprioception, pes cavus, macular dystrophy	Apraxia, adult-onset sensorineural hearing loss	Areflexia and decreased vibration sense in LL, vertical ophthalmoparesis

General Information	Family 9	Family 10			Family 11	Family 12	Family 13		Family 14	Family 15
	Patient 11	Patient 12	Patient 13	Patient 14	Patient 15	Patient 16	Patient 17	Patient 18	Patient 19	Patient 20
Sex/ethnicity	M/Europe	M/Europe	M/Europe	F/Europe	M/Europe	M/NA	M/Europe	F/Europe	F/Europe	M/Europe
Age at last examination, y	19	9	7	26	13	2	2	32	2	6
Genetic findings										
c.DNA	arr{hg38}9q34.11 (128,609,213-128,613,675)x1	c.4458delA			c.2197C>T	c.4936C>T	c.1879C>T		arr{hg38}9q34.11 (128591376-128600369)x1	c.2612del
Protein	p.(Ile1563_?)del (DEL2)	p.Lys1486Asnfs^∗^51			p.Arg733^∗^	p.Gln1646^∗^	p.Arg627^∗^		p.(Asp1003_Lys1193)del (DEL3)	p.Lys871Serfs^∗^5
Inheritance	De novo	AD			sporadic	De novo	AD		De novo	De novo
**Phenotypic category**	**DD/ataxia**	**DD**			**DD**	**DD**	**DD**		**DD**	**DD**
Initial symptoms	Ataxia	Speech delay	Speech delay	ID	ID	DD	Axial hypotonia	Axial hypotonia	DD	ID
Age of onset	15 mo	2 y	2 y	3 y	NA	Birth	5 mo	3 mo	9 mo	1 y
SZ (age of onset)	Febrile SZ	–	–	–	–	–	–	–	–	One febrile generalized tonic-clonic SZ (6 y)
Response to therapy	/	/	/	/	/	/	/	/	/	No therapy, controlled
EEG	NA	NA	NA	NA	NA	Increased excitability	NA	Normal	NA	Normal
Developmental history										
ID	+	Mild	Mild	–	Mild	+	+	–	?	–
Learning disability	+	+	+	+	+	+	+	+	?	Mild
Motor delay	+	–	–	–	–	+	+	+	+	Mild
Speech delay	+	+	+	–	–	+	+	NA	+	Moderate
Microcephaly	–	–	–		–	+	–	–	–	–
Neurologic findings										
Ataxia	Severe	–	–	–	–	–	+	+	–	–
Spasticity	–	–	–	–	–	–			–	–
Extensor planter reflex	–	–	–	–	–	–	–	–	–	–
Abnormal eye movement	Convergent strabismus	–	–	–	–	–	Nystagmus	Nystagmus	Strabismus	–
UL weakness	–	–	–	–	–	–	–	–	–	–
LL weakness	–	–	–	–	–	–	+	+	–	–
LL hyperreflexia	–	–	–	–	–	–	–	–	–	–
Ankle clonus	–	–	–	–	–	–	–	–	–	–
Bladder dysfunction	+	–	–	–	–	–	–	–	–	–
Amyotrophy	NA	–	–	–	–	–	–	–	–	–
Myoclonus	–	–	–	–	–	–	–	–	–	–
Brain MRI	Severe cerebellar atrophy and slightly dilated fourth ventricle	NA	NA	NA	NA	Nonspecific small gliosis in the right anterior border zone	Cerebellar atrophy	Severe cerebellar atrophy	NA	NA
Other clinical features	Pneumococcal meningitis aged 15 months, hypermetropia	Attention deficit Hyperactivity disorder	–	Basedow disease in remission	obesity; dyslipidemia; testicular hypoplasia (*MC4R* disease)	epicanthus, low-set ears, high philtrum, finger pads, sickle feet, sandal furrow	16p11.2 microdeletion involving *PRRT2*	–	hypotonia, poor motor planning and body awareness, tongue tie	obesity, constipation, mild myopia

General Information	Family 16	Family 17	Family 18	Family 19	Family 20	Family 21
	Patient 21	Patient 22	Patient 23	Patient 24	Patient 25	Patient 26
Sex/Ethnicity	F/Europe	M/NA	F/Europe	M/Europe	M/NA	M/Europe
Age at last examination, y	8	9	5	10	9	8
Genetic findings						
c.DNA	c.1127G>A	c.4390C>T	arr{hg38}9q34.11 (128,582,754-128,587,726)x1	c.6611G>A	c.4476del	arr{hg38}9q34.11 (128,587,422-128,600,316)x1
Protein	p.Trp376^∗^	p.Arg1464Trp	p.(Asn571_?)del (DEL4)	p.Arg2204Gln	p.Ala1493Argfs^∗^44	p.(Ala927_Lys1193) del (DEL5)
Inheritance	De novo	De novo	De novo	AD	Sporadic	De novo
**Phenotypic category**	**DD**	**DD/SZ**	**DD/SZ**	**DD/SZ**	**DD/SZ**	**DD/SZ**
Initial symptoms	Speech delay	SZ	Hypotonia	DD	ID	SZ
Age of onset	2 y	NA	2 mo	2 y	2 y	3 mo
SZ (age of onset)	–	Febrile and generalized myoclonic (3 y)	+ (2 y)	Myoclonic absence (5 y)	Absence and generalized tonic-clonic (6 y)	Infantile spasms (3 mo)
Response to therapy	/	NA	CBZ, controlled	NA	Refractory	Partially controlled
EEG	NA	NA	NA	NA	Typical absence, continuous spikes and waves during sleep aspect	Vertex sharp waves, right and left independent parietal waves during sleep
Developmental history						
ID	–	+	+	+	+	+
Learning disability	+	+	+	+	+	+
Motor delay	–	+	+	+	–	+
Speech delay	+	+	+	+	+	+
Microcephaly	+	+	–	–	–	+
Neurologic findings						
Ataxia	–	+	+	–	–	–
Spasticity	–	NA	–	–	–	–
Extensor planter reflex	–	–	–	–	–	–
Abnormal eye movement	–	–	Strabismus	–	–	–
UL weakness	–	NA	–	–	–	–
LL weakness	–	NA	–	–	–	–
LL hyperreflexia	–	–	–	–	–	–
Ankle clonus	–	–	–	–	–	–
Bladder dysfunction	–	–	–	–	–	–
Amyotrophy	–	–	–	–	–	–
Myoclonus	–	–	–	–	–	–
Brain MRI	Normal	NA	Mild cerebral atrophy	Normal	Normal	Mild delay in myelination
Other clinical features	Dyspraxia, hyperlaxity	–	Hypotonia, sleep problems, constipation	–	ADHD	Poor proprioception, hypotonia

General Information	Family 22	Family 23	Family 24	Family 25	Family 26
	Patient 27	Patient 28	Patient 29	Patient 30	Patient 31
Sex/ethnicity	F/Europe	M/Europe	F/Africa	F/Europe	M/India
Age at last examination, y	16	12	20	7	1
Genetic findings					
c.DNA	c.6247_6249del	c.6811G>A	c.6619_6621del	c.4344G>A	c.6908_6916dup
Protein	p.Lys2083del	p.Glu2271Lys	p.Glu2207del	p.Gln1448= (splice) exon 38	p.Asp2303_Leu2305dup
Inheritance	Unknown	De novo	De novo	Unknown	De novo
**Phenotypic category**	**DEE**	**DEE**	**DEE**	**DEE**	**DEE**
Initial symptoms	SZ	SZ	Hypotonia	Axial hypotonia	SZ
Age of onset	2 mo	4 mo	8 mo	Birth	Birth
SZ (age of onset)	Absence SZ (2 mo)	Myoclonic jerks, dystonic spasms (4 mo)	Tonic, oral automatisms, upward deviation of gaze	West syndrome (4 mo)	Generalized tonic-clonic (birth)
Response to therapy	NA	Refractory	Controlled	Refractory	Refractory
EEG	NA	Slow-wave activity, loss of normal rhythms, temporal spike, sharp wave discharges	Diffuse slow activity, irregular low to medium amplitude	Hypsarrhythmia	Multifocal epilepsy
Developmental history					
ID	+	+	+	severe	NA
Learning disability	+	+	+	severe	NA
Motor delay	+	+	+	severe	severe
Speech delay	+	+	+	severe	NA
Microcephaly	–	+	+	+	severe
Neurologic findings					
Ataxia	+	–	–	NA	NA
Spasticity	–	–	+	NA	+
Extensor planter reflex	–	–	+	–	+
Abnormal eye movement	Strabismus	–	Strabismus	Nystagmus	Nystagmus
UL weakness	–	–	+	+	+
LL weakness	–	–	+	+	+
LL hyperreflexia	–	+	–	–	+
Ankle clonus	–	–	–	–	+
Bladder dysfunction	–	–	–	–	–
Amyotrophy	–	–	+	–	–
Myoclonus	–	–	–	–	–
Brain MRI	NA	Cerebellar atrophy, delayed myelination, thin corpus callosum	NA	Delayed myelination, progressive brain and pontocerebellar atrophy	Delayed myelination, cerebral atrophy, thin corpus callosum
Other clinical features	–	Rod cone retinal dystrophy, GERD, scoliosis, dislocated hip	Visual impairment, dysmorphism, scoliosis	–	–

All variants are reported with reference to RefSeq NM_001130438.3.

*AD*, autosomal dominant; *ADHD*, attention deficit hyperactivity disorder; *CBZ*, carbamazepine; *DD*, developmental delay; *DEE*, developmental epileptic encephalopathy; *EEG*, electroencephalography; *F*, female; *GERD*, gastroesophageal reflux; *HA*, hereditary ataxia; *HSP*, hereditary spastic paraplegia; *ID*, intellectual disability; *LL*, lower limbs; *M*, male; *MRI*, magnetic resonance imaging; *NA*, not available; *SZ*, seizures; *UL*, upper limbs; *VAL*, valproic acid; /, not applicable; –, absence of manifestation; +, presence of manifestation.

By screening DECIPHER database,^8^ 4 additional variants were identified; 1 missense variant in patient 22 and 3 de novo microdeletions in the *SPTAN1* gene. DEL2 in patient 11 is a 4.46-kilobase deletion that removes exons 36 to 40. This patient presented with ataxia and severe DD. Patients 23 and 26, who carried DEL4 (exons 14-20) and DEL5 (exons 20-27), respectively, presented with DD and seizures.

A total of 13 additional *SPTAN1* families were identified through GeneMatcher.^9^ Patients 5 and 6 shared the same de novo missense variant, p.(Arg19Trp), and HSP phenotype as patients 1 to 4. Nevertheless, patient 5 presented with complex HSP, learning disability, and seizures. Three frameshift variants were identified in 5 patients (patients 12-14, 20, and 25) with DD/Seizures. Dominant inheritance was noted in 3 patients (patients 12-14). Five patients (patients 15-18 and 21) with ID/seizures carried nonsense *SPTAN1* variants. The last variant was an in-frame deletion (DEL3), which was identified in patient 19, a 2-year-old presented with DD. This deletion is almost 9 kilobase, encompasses exons 22 to 27, and overlaps with DEL5. All variants reported in this study were classified according to the guidelines of the American College of Medical Genetics and Genomics and Association for Molecular Pathology.^17^ (Supplemental Table 2).

### Clinical phenotypes

Detailed clinical information was collated for 31 individuals from 26 unrelated families carrying heterozygous variants in *SPTAN1* (Table 1). Common phenotypes including ID/learning disability and motor delay were reported in 73.5% and 58.8% of our cohort, respectively. Remarkably, around half of the patients (15/31) manifested ataxia, and seizures were reported in almost one-third of the cases (12/31).

Recently reported phenotypes of HSP/HA were identified in a subgroup of our patients. A total of 8 individuals from 6 families presented with pure HSP/HA and further 2 families with complex HSP (Supplemental Video 1). All patients with HSP showed typical features. Most of them had lower limb hyperreflexia and/or ataxia (6/8) whereas none had sensory abnormalities. In contrast, 2 patients with HA showed a pure phenotype. Abnormal eye movement, a common condition in patients with ataxia, was noted in 70% of this HSP/HA group. Severe phenotype of DEE was reported in 5 patients. All had seizures in early months of life and had severe DD. The remaining 16 patients from 13 families presented with varying degrees of DD.

### Functional consequences of *SPTAN1* variants in patient-derived fibroblasts

**Figure 3:**
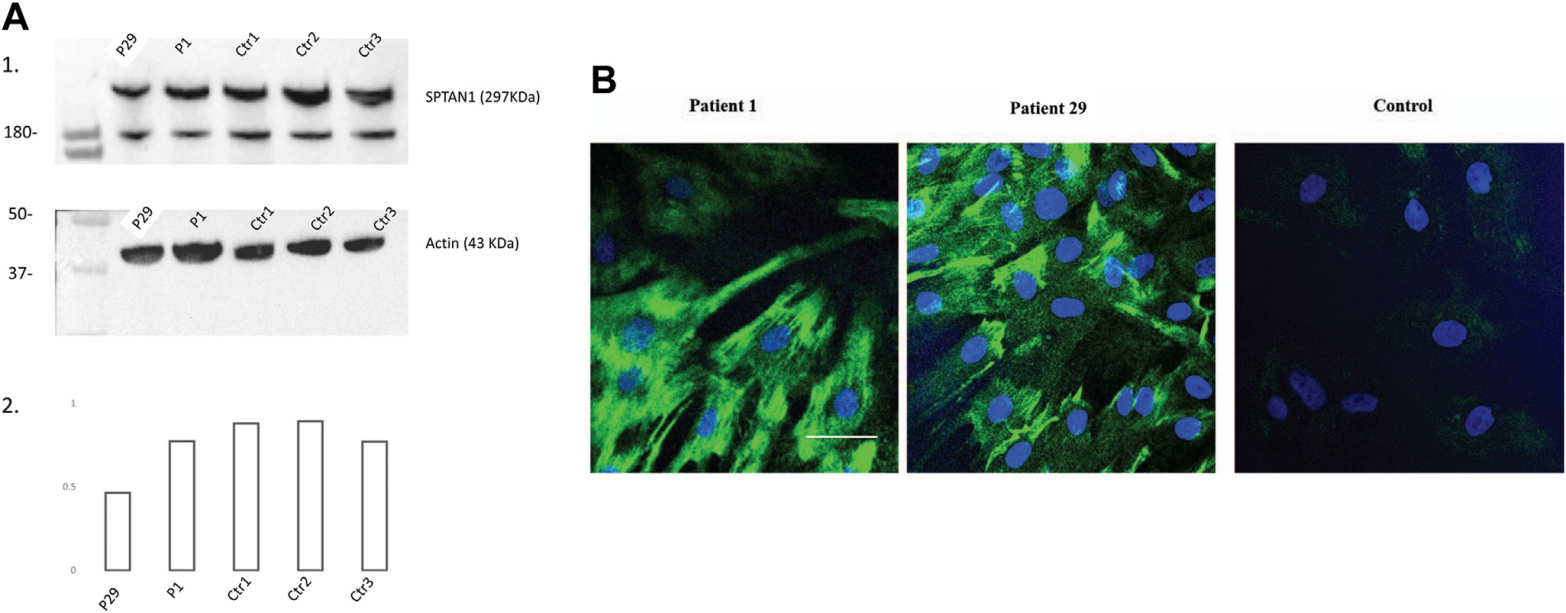
**Representative images of αII**-**spectrin protein expression and staining pattern in fibroblast cells derived from 2 patients and unrelated controls.** A. Western blot. 1. Western blotting of protein extracted from fibroblast cell lines of patients 1 and 29 and 3 wild-type age-matched controls. 2. Densitometric analysis of western blot using BioRad Image Lab software after relative normalization to actin as a housekeeping protein. The analysis showed no change in protein expression in patient 1 but showed a quantitative reduction of protein expression in patient 29. B. Immunocytochemical staining of αII-spectrin expression in primary fibroblasts of patients 1 and 29 and unrelated control individual with Alexa Fluor 488 conjugated secondary antibody (green) and Hoescht 33342 nuclear staining (blue). Scale bar represents 50 μm. Immunocytochemical staining showed high immunofluorescence brightness and intense immunoreactivity and aggregation of αII-spectrin in both studied patients compared with the healthy unrelated control. Ctr, control.

We analyzed fibroblasts derived from 2 patients with the missense variants p.(Arg19Trp) and p.(Glu2207del) (Figure 3). Although western blot did not show a quantitative difference in protein expression between patient 1 and controls, a quantitative reduction of protein expression in patient 29 was noted. Nevertheless, a high immunofluorescence brightness and intense immunoreactivity of αII-spectrin was observed in both the studied patients. In patient 1, αII-spectrin was ubiquitously expressed throughout the fibroblast cells, whereas there was a more localized aggregation of αII-spectrin in the plasma membrane with a relatively higher immunofluorescence brightness observed in the fibroblasts of patient 29.

### Structural modeling of *SPTAN1* missense variants

The effect of missense variants on αII-spectrin protein structure (Q13813-1) was investigated using homology modeling of experimentally validated models^12^, ^13^, ^14^ (Supplemental Figure 2). Variant p.(Arg19Trp) had the most deleterious effect on protein structure. This variant is located within the N-terminal tetramerization domain and results in steric clashes with 2 leucine residues in the beta chain. Less severe effect was noted in modeling of p.(Arg1464Trp) and p.(Arg2204Gln) variants. Structural modeling showed no structural effect for p.(Glu2271Lys), p.(Arg2124Cys), and p.(Ser2448Phe); however these would likely affect αII-spectrin heterodimerization with its partner, β-spectrin. Overall, most (4/6) of the reported missense variants led to protein destabilization (Supplemental Table 3). In addition, according to in silico pathogenicity prediction, almost all missense variants identified in this study are potentially pathogenic (Supplemental Table 4).

## Discussion

Identification of an enrichment of *SPTAN1* heterozygous variants in patients presenting with HA and HSP confirms *SPTAN1* involvement in a wide phenotypic spectrum. We suggest that *SPTAN1* is a genetic cause of neurodevelopmental disorders, with 3 phenotypic subgroups (Table 1). The first group comprises patients with DEE presenting with severe phenotype (OMIM 613477). DEE was identified in 5 families, consistent with previous reports.^2^ A total of 16 patients manifested milder phenotype of DD with or without childhood-onset seizures forming the second phenotypic group. The final group consists of patients (*n* = 10) with pure or complex HSP/HA.

Involvement of *SPTAN1* variants in peripheral nervous system abnormalities was previously reported for heterozygous variants causing hereditary motor sensory neuropathy^3^ and biallelic variants associated with autosomal recessive HSP.^4^ The phenotype of HA and HSP is further supported by a previously reported *SPTAN1* mouse model presenting with unsteady gait and spasticity^5^ (Supplemental Video 2) and the recently published study reporting de novo and dominant variants of *SPTAN1* in patients with ataxia and patients with spastic paraplegia.^18^

Phenotypic heterogeneity may be explained by the involvement of *SPTAN1* pathogenic variants in different mechanisms of pathogenicity as it was described with other structural proteins.^19^ Syrbe et al^2^ concluded that variants in the last 2 αII-spectrin repeats are associated with severe phenotype due to protein aggregation with dominant negative effect. This mechanism is supported by our observation in αII-spectrin immunocytochemistry experiment performed on fibroblast of patient 29. However, further experiments on multiple cell lines would be imperative to support this hypothesis.

We noted that the excess of truncating variants in the milder category of our cohort (DD +/– seizures) is in agreement with the proposed mechanism of quantitative defect of αII-spectrin protein leading to a milder phenotype.^20^ We suggest that truncating variants are responsible for a mild DD with or without epilepsy.

In our HSP/HA group, we have detected accumulation of αII-spectrin in fibroblasts of patient 1 with the recurrent p.(Arg19Trp) variant, indicating abnormal protein function. Such interesting finding adds further evidence to Van de Vondel et al^18^ report of the recurrent p.(Arg19Trp) variant detected in 7 families with spastic paraplegia. All other identified variants in this group of our patients were missense variants except for 1 splice altering variant. All are predicted to have a moderate protein effect except for p.(Arg19Trp), which is localized at an essential position of the N-terminal tetramerization domain.^1^ An arginine to tryptophan change, p.(Arg35Trp), at the N-terminus has been reported previously in erythrocytic α-spectrin gene (*SPTA1*), where it prevented N-terminal domain of α-spectrin to form heterotetramers with its beta partner. We suggest a similar mechanism for *SPTAN1* variant, p.(Arg19Trp).^21^ In a previous report, we showed that ataxia and/or HSP cases may be accounted by hypomorphic pathogenic variants in genes known to manifest with severe phenotypes when mutated.^22^ The p.(Arg2124Cys) and p.(Ser2448Phe) variants identified in families 5 and 6 are missense variants that are predicted to introduce mild structural alterations in the αII-spectrin protein potentially explaining the milder neurologic impairment. In family 8, with mild and late-onset ataxia, a splice altering variant (c.3519+2T>G) was detected. The resulting predicted in-frame deletion rather than a loss of function might explain the mild neurologic phenotype in this family.

It is interesting to notice that αII-spectrin forms heterotetramers with each of the 4 nonerythrocytic β-spectrins 1 to 4 at precise membrane domains.^23^ The first 3 β-spectrin genes (*SPTBN1*, *SPTBN2*, and *SPTBN4*) are responsible of multiple neurologic disorders, depending on the gene and inheritance pattern.^19^ Particularly, βIII spectrin (*SPTBN2)* is specific to Purkinje cells and is involved in a relatively pure late-onset ataxia phenotype.^24^ αII-Spectrin broad distribution in the neurons is therefore a plausible explanation of the pleiotropic consequences of *SPTAN1* variants.

Molecular diagnosis of patients with neurodevelopmental disorders is often challenging owing to both phenotypic and genetic heterogenicity. We were able to expand the phenotypic and genetic spectrum of *SPTAN1* variants, shedding light on the critical role that αII-spectrin has in maintaining brain health.

## Data Availability

De-identified data are available upon request. Data requests can be made via email to the corresponding author and will be pending data use agreements.

## Conflict of Interest

The authors declare no conflicts of interest.

## Members of The Genomics England Research Consortium

J.C. Ambrose, P. Arumugam, E.L. Baple, M. Bleda, F. Boardman-Pretty, J.M. Boissiere, C.R. Boustred, H. Brittain, M.J. Caulfield, G.C. Chan, C.E.H. Craig, L.C. Daugherty, A. de Burca, A. Devereau, G. Elgar, R.E. Foulger, T. Fowler, P. Furió-Tarí, J.M. Hackett, D. Halai, A. Hamblin, S. Henderson, J.E. Holman, T.J.P. Hubbard, K. Ibáñez, R. Jackson, L.J. Jones, D. Kasperaviciute, M. Kayikci, L. Lahnstein, K. Lawson, S.E.A. Leigh, I.U.S. Leong, F.J. Lopez, F. Maleady-Crowe, J. Mason, E.M. McDonagh, L. Moutsianas, M. Mueller, N. Murugaesu, A.C. Need, C.A. Odhams, C. Patch, D. Perez-Gil, D. Polychronopoulos, J. Pullinger, T. Rahim, A. Rendon, P. Riesgo-Ferreiro, T. Rogers, M. Ryten, K. Savage, K. Sawant, R.H. Scott, A. Siddiq, A. Sieghart, D. Smedley, K.R. Smith, A. Sosinsky, W. Spooner, H.E. Stevens, A. Stuckey, R. Sultana, E.R.A. Thomas, S.R. Thompson, C. Tregidgo, A. Tucci, E. Walsh, S.A. Watters, M.J. Welland, E. Williams, K. Witkowska, S.M. Wood, M. Zarowiecki
