# Supplementary material for: Expanding SPTAN1 monoallelic variant associated disorders: From epileptic encephalopathy to pure spastic paraplegia and ataxia
Source: Genet Med. 2023 Jan;25(1):76–89. doi: 10.1016/j.gim.2022.09.013 (PMC10620943; doi:10.1016/j.gim.2022.09.013)
Supplement: Supplementary data [file mmc1.pdf]

SUPPLEMENTAL DATA

1. **Supp Method:** Western blot - Immunocytochemistry and confocal microscopy.

2. **Case studies**

3. **Tables :**

Table S1: *SPTAN1* probably damaging variants in the 100K GP cohorts
Table S2: *SPTAN1* (NM_001130438.3) variants reported in the study and in silico prediction score.
Table S3: DynaMut prediction outcomes for the missense variants identified in the study.
Table S4: Insilico predictions of missense SPTAN1 (NM_001130438.3) previously reported variants

4. **Figures**

Figure S1: IGV for *SPTAN1* gene deletion in family 7.
Figure S2: Structural impact of *SPTAN1* missense variants

5. **Videos**

Video S1: Phenotype of patient 1 with the recurrent *SPTAN1*; p. Arg19Trp variant, exhibiting a spastic and scissoring gait.
Video S2: Ataxic phenotype of nine-month-old C57BL/6J mouse with p. Arg1098Gln variant showing obvious motor and coordination difficulty.

Supp Method:

Western blot

Western blot analysis was performed on extracting protein from fibroblasts derived from Patient 1 and 29. Three control proteins were extracted from control fibroblast cell lines from healthy, unrelated individuals (3 years old, 24 years old and 26 years old). Cells were lysed in radioimmunoprecipitation assay buffer [150 mM NaCl, 1.0% IGEPAL® CA-630, 0.5% sodium deoxycholate, 0.1% SDS, 50 mM Tris (Sigma)], to which Protease Inhibitor Tablets were added to avoid proteolytic degradation (Pierce Protease Inhibitor Tablets, Thermoscientific), for 1 hr in ice, vortexing intermittently, followed by centrifugation at 14,600 rpm for 10-15 minutes at 4° C to retrieve the supernatants. Total protein concentrations were determined by Bicinchoninic acid protein assay (PierceTM). Protein lysates were diluted 1:1 with Nu PAGE LDS sample buffer, and resolved by % PAGE, transferred onto nitrocellulose membrane and incubated in blocking solution [Phosphate-Buffered Saline (PBS), 3% ECL blocking agent, 0.1 % Tween-20] for 1 h at room temperature, followed by overnight incubation with primary antibody at 4º C [anti-SPTAN1, 1:1000 dilution; abcam D8B7, ab11755; Anti-Actin, Sigma,1 1:5000 dilution]. Membranes were washed with PBS-T [Phosphate-Buffered Saline, 0.1% Tween-20] five times for five minutes followed by incubation with secondary antibody (HRP conjugated anti-mouse) for 1hr at room temperature. Blots were visualized by chemiluminescence using the Amersham ECL Prime Substrate [ECL Prime Luminol Enhancer Solution, ECL Primer Peroxide Solution, 1:1 dilution]. Results were analysed with a densitometric analysis using BioRad Image LabTM software and "lane and bands" tool. Calculation was done using a relative relationship method. Actin was used as housekeeping protein. The adjusted volumes of SPTAN1 bands were normalized over the adjusted volume of beta-actin.

Immunocytochemistry and confocal microscopy

Primary fibroblasts of Patients 1 and 29 were primarily seeded onto sterilised glass coverslips for 48 hr in a 6-well plate. Post treatments, cells were fixed with 4% paraformadehyde (PFA) (Sigma-Aldrich, F8775) for 20 minutes. After 3x phosphate buffered saline (PBS) washes, cells were treated with blocking and permeabilization solution (10% FBS, 0.25% Triton-X-100 in PBS) at room temperature (RT) for 30 minutes. Cells were then incubated at 4°C overnight in antibody solution (10% FBS in PBS) containing primary mouse anti-SPTAN1 antibody (1:200 dilution; D8B7, ab11755), detecting αII- spectrin. After 3x PBS washes, cells were incubated with goat anti-mouse 488 Alexa Fluor secondary antibody (1:2000 dilution; Thermo Fisher Scientific, A11029), and Hoescht 33342 (1:2000 dilution; Thermo Fisher Scientific, 62249) nuclear staining in 10% FBS/PBSfor 1 hr at RT. After extensive washing in PBS, cells were embedded and cover-slipped in mounting solution (Dako; Agilent Technologies). The resultant immunostaining was observed using a 40x oil immersion objective on a Zeiss LSM 710 confocal microscope and representative Z-stack images were acquired using the Zen 2009 imaging software (Zeiss).

Case studies

Patient 1 is a 41-years-old lady diagnosed with HSP at 12 years old. At the age of 8, she presented with walking difficulties and frequent falls, slowly progressing leg weakness and stiffness. By the age of 15, crutches were required for ambulation. Currently, she presents with a spastic and scissoring gait, and she can walk with assistance for 30 minutes. She also reports occasional urinary and fecal urgency and incontinence. At neurological examination, there is bilateral lower limb hypertonia and muscle weakness (MRC grade 0 in bilateral hip flexion, 2 in hip abduction, 3 on knee extension and 0 on knee flexion), and hyperreflexia with positive crossed abductor jerks. No impairment is documented regarding vibration, joint position and temperature sense. She never experienced seizures and her cognitive function is not impaired. Brain MRI is unremarkable.

Patient 11 is a 19 year old gentleman that was first noted to be ataxic during an admission to hospital at 15 months of age, with pneumococcal meningitis presenting with a febrile seizure. Prior to this episode of meninigitis, the patient had mild developmental delay. Ataxic symptoms progressed in early childhood and at the age of 4 were assessed as severe. There was progressive significant truncal limb ataxia with no associated weakness. The patient has severe global developmental delay and intellectual disability. There was three febrile seizures in early childhood. The patient was educated in a special needs school and as a young adult continues to require assistance with all activities of daily living. He is able to mobilise with a 4 wheeled walking frame with the assistance of one person. He can communicate in basic sentences but speech can be difficult to understand due to dysarthria. He is able to recognise letters the alphabet, but is not able to read, write or hold a pen. He is able to feed himself with a spoon, and wears a nappy for toileting. The patient interests are said to be aligned to that of a 4 year old. MRI brain scan at the age of 4 identified severe cerebellar atrophy and a slightly dilated 4th ventricle. Imaging was repeated at the age of 7 and showed no interval changes. Extensive neurometabolic investigations were unremarkable.

Table S1: *SPTAN1* probably damaging variants in the 100K GP cohorts Cases were defined as all 100K GP[(1)] probands recruited under hereditary ataxia/hereditery spastic paraplegia , while 'controls' were all remaining individuals recruited in the 100K GP except those recruited under neurological and neurodevelopmental disorders or metabolic disorders. Variants were defined as rare (MAF $< 1X\ 10^{-5}$) and either protein-truncating variants (essential splice site, frameshift, and nonsense) or missense variants predicted to be pathogenic by 2 in-silico tools (CADD[(2)], Polyphen [(3)]).

**Rare *SPTAN1* variants**	**Cases (HSP/HA)**	**Controls**
Yes	12	52
No	1,130	23,795
Total	1,142	23,847

Table S2: *SPTAN1* (NM_001130438.3) variants reported in the study and in silico prediction score. hg38 positions, exons and domains localization are given. CADD score (phred) [(2)] are given for missenses, nonsenses and splice variant. *In silico* prediction score SIFT [(4)] (0: damaging,1:benign) and PolyPhen2 [(3)] (1:damaging,0:benign) were used for missenses, MaxEnt (positive if delta > 10%) and splice AI(splice-altering if > 0.2) for splicing variant. Classification of variants was performed according to the guidelines of the American College of Medical Genetics.[(5)] DL: splice donor loss, wt:wild, P: Pathogenic, LP: Likely Pathogenic, VUS: Variant of unknown significance, D:Damaging, B:Benign, E: Exon.

Family	Seg	Variant class	Position (hg38;chr9)	E	Codon change	Amino acid change	Domain	CADD	In silico prediction		ACMG criteria	ACMG classification
1,2,3,4	*denovo AD*	missense	128566795	2	c.55 C>T	**p.Arg19Trp**	Repeat 0, Helix C	26.6	SIFT: D	PolyPhen2:D	PS1,PS2,PS3,PM2, PP2, PP3	P
16	denovo	nonsense	128578151	9	c.1127G>A	**p.Trp376***	Repeat 4, Helix A	38.0	/	/	PVS1,PS2,PM2	P
18	denovo	deletion (DEL4)	128,582,754-128,587,726	14-20	c.1711-?	**p.571-?**	Repeat 4, Helix C	/	/	/	PVS1_strong, PS2, PM4	LP
13	AD	nonsense	128583149	15	c.1879C>T	**p.Arg627***	Repeat 6, Helix B	38.0	/	/	PVS1, PM2,PP1	LP
11	sporadic	nonsense	128584285	17	c.2197C>T	**p.Arg733***	Repeat 7, Helix B	38.0	/	/	PVS1,PM2,PM6	P
15	denovo	frameshift	128585796	19	c.2612del	**p.Lys871Serfs*5**	Repeat 8, Helix C	/	/	/	PVS1,PS2,PM2	P
21	denovo	deletion (DEL5)	128,587,422-128,600,316	19-25	/	**p.(Ala927_Lys1193del)**	Repeat 9, Helix B - SH3 domain	/	/	/	PVS1_moderate, PS2,PP4	LP
14	denovo	deletion (DEL3)	128591376-128600369	22-27	/	**p.(Asp1003_Lys1193)del**	Repeat 10, linker, SH3 domain	/	/	/	PVS1_moderate, PS2	LP
7	sporadic	deletion (DEL1)	128,597,905-128,602,892	24-25	/	**p.(Asp1139_Lys1193)del**	Repeat 10, Helix B SH3 domain	/	/	/	PVS1_moderate	VUS
8	sporadic	splice	128600117	25	c.3519+2T>G	**p.?**	Intronic	33.0	MaxEnt: wt:10.28 --> 2.6	SpliceAI: DL(1)	PVS1_moderate, PP3	VUS
25	unknown	synonymous splice	128608049	33	c.4344G>A	**Q1448=**	Repeat 13, Helix A	24.1	MaxEnt: wt:6.99 --> 3.84	SpliceAI: DG(0.55)	PP4	VUS
17	denovo	missense	128608175	34	c.4390C>T	**p.Arg1464Trp**	Repeat 13, Helix A	26.3	SIFT: D	PolyPhen2: B	PS2,PM2,PP2,PP3	LP
10	AD	frameshift	128608243	34	c.4458delA	**p.Lys1486Asnfs*51**	Repeat 13, Helix B	/	/	/	PVS1,PM2,PP1	P
20	sporadic	frameshift	128608261	34	c.4476del	**p.Ala1493Argfs*44**	Repeat 13, Helix B	/	/	/	PVS1,PM2	P
9	denovo	deletion (DEL2)	128,609,213-128,613,675	35-40	c.4687-?	**p.1563-?**	Repeat 14, Helix B	/	/	/	PVS1_strong, PS2	LP
12	denovo	nonsense	128612139	39	c.4936C>T	**p.Gln1646***	Repeat 14, Helix C	39.0	/	/	PVS1,PS2,PM2	P
22	unknown	inframe del	128625940	48	c.6247_6249del	**p.Lys2083del**	Repeat 19, Helix C	/	/	/	PM2,PM4	VUS

5	sporadic	missense	128626481	49	c.6370C>T	**p.Arg2124Cys**	Repeat 19, A/B loop	34.0	SIFT: D	PolyPhen2: D	PM2,PP2,PP3	VUS
19	AD	missense	128627420	50	c.6611G>A	**p.Arg2204Gln**	Repeat 19, Linker	35.0	SIFT: D	PolyPhen2: D	PM1,PM2,PP1,PP2,PP3	LP
24	denovo	inframe del	128627423	50	c.6619_6621del	**p.Glu2207del**	Repeat 19, Linker	/	/	/	PS1,PS2,PS3,PM2, PM4,PP4	P
23	denovo	missense	128632175	53	c.6811G>A	**p.Glu2271Lys**	Repeat 20, Helix B	33.0	SIFT: D	PolyPhen2: D	PS1,PS2,PM2,PP2,PP3, PP4	P
26	denovo	inframe dup	128632271	53	c.6908_6916dup	**p.Asp2303_Leu2305dup**	EF-Hand I	/	/	/	PS1, PS2, PS3, PM2, PM4, PP4	P
6	unknown	missense	128633243	56	c.7343C>T	**p.Ser2448Phe**	EF-Hand II	25.1	SIFT: D	PolyPhen2: B	PP2,PP3	VUS

Table S3: **DynaMut prediction outcomes for the missense variants identified in the study.** DynaMut [(6)] insilico tool was used for prediction of protein stability based on the following models; p.Arg19Trp: 3F31[(7)], p.Arg1464Trp: 3fb2[(8)], p.Arg2124Cys: predicted template from Phyre2 [(9)] based on crystal structure of repeats 15 and 16 of chicken brain2 alpha spectrin (1u5p-A), p.Arg2204Gln and p.Glu2271Lys: predicted template from Phyre2 based on crystal structure of the rod domain of alpha-actinin (1hci-B), p.Ser2423Phe : Phyre build on cryoEM structure of chicken gizzard smooth muscle alpha-actinin (1sjj-B).

**Missense variant**	**ΔΔG prediction outcome**	**Δ Vibrational Entropy Energy Between Wild-Type and Mutant(ΔΔS_{Vib} ENCoM)**
**p.Arg19Trp**	-0.674 kcal/mol (Destabilizing)	0.095 kcal.mol-1.K-1 (Increase of molecule flexibility)
**p.Arg1464Trp**	1.242 kcal/mol (Stabilizing)	-4.870 kcal.mol-1.K-1 (Decrease of molecule flexibility)
**p.Arg2124Cys**	-0.936 kcal/mol (Destabilizing)	1.717 kcal.mol-1.K-1 (Increase of molecule flexibility)
**p.Arg2204Gln**	-0.237 kcal/mol (Destabilizing)	0.233 kcal.mol-1.K-1 (Increase of molecule flexibility)
**p.Glu2271Lys**	-0.538 kcal/mol (Destabilizing)	-0.056 kcal.mol-1.K-1 (Decrease of molecule flexibility)
**p.Ser2448Phe**	0.676 kcal/mol (Stabilizing)	-0.118 kcal.mol-1.K-1 (Decrease of molecule flexibility)

Table S4: Insilico predictions of missense *SPTAN1* (NM_001130438.3) previously reported variants. Conservation study of *SPTAN1* throughout Metazoa was predicted “High” if the same amino acid in conserved in most Cnidaria and Lophotrochozoa species; “Moderate” if the amino acid of the same family is found in most Cnidaria and Lophotrochozoa species; and “Low” if the amino acid is poorly conserved in most Cnidaria and Lophotrochozoa species.Variants reported in the current study are in bold.

HGVSc	HGVSp	CADD	SIFT	PolyPhen2	Conservation (metazoa)	Grantham score	References
c.55C>T	**p.Arg19Trp**	25,8	0,0 D	1,0 D	High	101	Von de Vondel, 2022 ; **Morsy, 2022**
c.271G>A	p.Glu91Lys	32	0,003 D	1,0 D	Moderate	56	Gilissen, 2014
c.362G>T	p.Arg121Lys	30	0,01 D	0,401 B	Low	26	An, 2014
c.533G>A	p.Gly178Asn	27,2	0,001 D	0,992 D	High	80	Syrbe, 2017
c.917C>T	p.Ala306Val	24	0,292 T	0,998 D	High	64	Syrbe, 2017
c.1303T>G	p.Ser435Ala	22,9	0,297 T	0,245 B	Low	99	Bagnall, 2014
c.1697G>C	p.Arg566Pro	29,9	0,002 D	0,997 D	Moderate	103	Hamdan, 2012
c.2572G>T	p.Ala858Ser	20,8	0,307 T	0.0 B	Moderate	99	Leveille, 2019
c.3292C>T/A	p.Arg1098Cys/Ser/Gln	31	0,002 D	1,0 D	High	180	Miazek 2020, Hernandez 2021, Von de Vondel 2022
c.3716A>G	p.His1239Arg	27,3	0,01 D	0,96 D	Moderate	29	Syrbe, 2017
c.4162A>G	p.Ile1388Val	24,1	0,034 D	0,498 B	High	29	Xie, 2021
c.4283C>G	p.Ala1428Gly	21,6	0,078 T	0,004 B	Low	60	Bobbili, 2014 ; Leveille, 2019
c.4390C>T	**p.Arg1464Trp**	24,7	0,0 D	0,012 B	Moderate	101	**Morsy, 2022**
c.4828C>T	p.Arg1610Trp	25	0,0 D	0,109 B	High	101	Syrbe, 2017
c.4846G>A	p.Asp1616Asn	23,1	0,704 T	0,045 B	Low	23	Terrone, 2020
c.4870C>T	p.Arg1624Cys		0,021 D	0,998 D	Low	180	Von de Vondel, 2022
c.5326C>T	p.Arg1776Trp	28,2	0,001 D	1,0 D	Moderate	101	Syrbe, 2017
c.5936A>G	p.Glu1979Gly	32	0,002 D	0,979 D	Moderate	98	Von de Vondel, 2022
c.6184C>T	p.Arg2062Trp	28,5	0,0 D	1,0 D	High	101	Syrbe, 2017
c.6370C>T	**p.Arg2124Cys**	29,9	0,001 D	1,00 D	High-	180	**Morsy, 2022**
c.6611G>A	**p.Arg2204Gln**	32	0,00 D	0,997 D	High-	43	**Morsy, 2022**
c.6614A>C	p.Gln2205Pro	24,4	0,00 D	0,232 B	High-	76	Von de Vondel, 2022
c.6811G>A	**p.Glu2271Lys**	32	0,004 D	0,995 D	High	56	**Morsy, 2022**
c.6927G>C	p.Met2309Ile	27,6	0,007 D	0,656 PD	High	10	Leveille (2019)
c.6947A>C	p.Gln2316Pro	29,7	0,002 D	0,96 D	High	76	Stavropoulos (2016)
c.7343C>T	**p.Ser2448Phe**	28,4	0,019 D	0,994 D	Low	155	**Morsy, 2022**

Supp. Figures :

Figue S1: IGV for *SPTAN1* gene deletion in family 7.
IVG_2.4.4 visualization of deleted regions identified in family 7 (patient 9; DEL1: arr{hg38}9q34.11(128,597,905-128,602,892)x1)

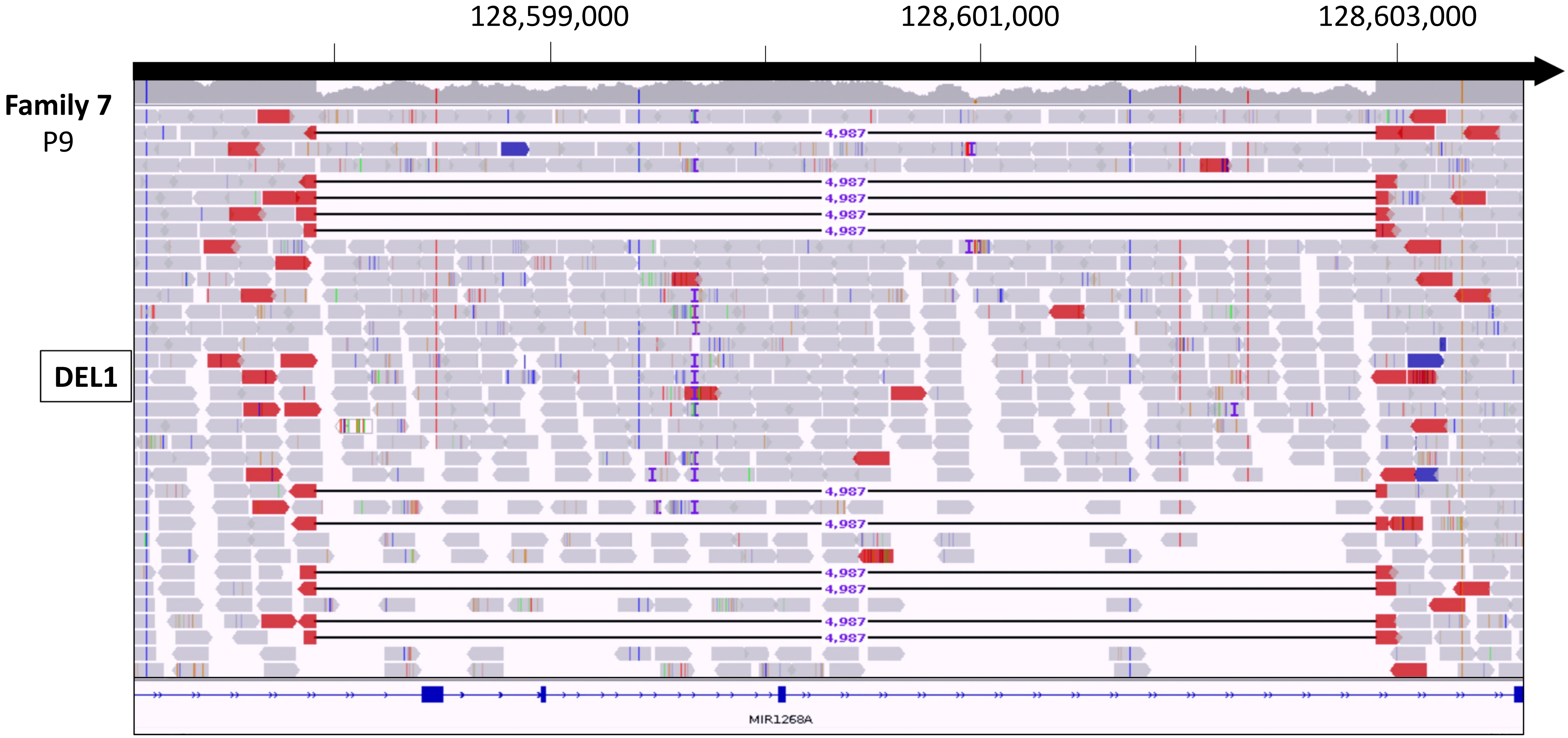

Family 7
P9
DEL1
128,599,000
128,601,000
128,603,000
4,987
MIR1268A

Figure S1: Structural impact of *SPTAN1* missense variants.

Model of human spectrin protein shown in UCSF Chimera software. For each missense variant: the model used for simulation is on the top with the position of variant highlighted in red, the wild-type (left) and mutated (right) amino acid and their surroundings are shown in close-ups. The position of each disease-associated variant is highlighted in red. Affected residues and neighbouring residues within a 5.0-Å range are shown as sticks and side chains are coloured by element (carbon = grey, oxygen = red, nitrogen = blue, hydrogen = white). Hydrogen bonds as identified by UCSF Chimera built-in tools are represented by light blue lines. Steric clashes and pseudo-bonds as identified by UCSF Chimera built-in tools are represented by red and yellow lines, respectively. [(10)] Orthologous gene conservation through several metazoa species are shown for affected and neighbouring residues with correpsonding EVE Visual score [(11)] (red: pathogenic; white: benign). (H.s: Homo sapiens; D.m: Drosophila melanogaster; O.b: Octopus bimaculoides; O.f: Orbicella faveolata; E.d: Exaiptasia diaphana T.a: Trichoplax adherens).

A) R19W: Simulation of p. Arg19Trp missense variant based on crystal structure of the N-terminal region of αII-spectrin Tetramerization Domain (3F31).[(7)] Variant causes multiple disruption as it eliminates 2 hydrogen bonds with neighbouring isoleucine at position15 and Glutamine at position 147 of B chain. Also, it induces steric clashes with Leucine residues at positions 78 and 146 of B chain.

B) R1464W: Analysis was based on the Crystal structure of the human brain alpha spectrin repeats 15 and 16 (3fb2).[(8)] Arginine at position 1464 is located within the A-helix of Spectrin repeat number 13. The change of Arginine to Tryptophan is predicted to cause moderate impact with 36 steric clashes with neighbouring Phenylalanine and Leucine residues at positions 1467 and 1536, respectively.

C) R2204Q: 3D Modelling done on predicted template from Phyre2 [(9)] based on crystal structure of the rod domain of alpha-actinin (1hci-B), with 99% of the sequence modelled with 99.8% confidence. Substitution of Arg2204 by Glutamine in spectrin repeat 20 is predicted to cause moderate disruption, with introduction of 16 steric clashes with adjacent Leucine at position 2119.

D) E2271K: The same model as R2204Q was used. However, the Substitution of Glutamic acid 2271 in spectrin repeat 20 by Lysine is predicted to be tolerated, with no steric clashes.

E) R2124C: 3D Modelling done on predicted template from Phyre2 [(9)] based on crystal structure of repeats 15 and 16 of chicken brain2 alpha spectrin (1u5p-A), with 90% of sequence modelled with 99.9% confidence. This substitution is tolerated since it eliminates hydrogen bonds with adjacent Aspartate residue at position 2284 and do not introduce streric clashes.

F) S2448F: Modelling based on template generated by Phyre build on cryoEM structure of chicken gizzard smooth muscle alpha-actinin (1sjj-B), with 93% of the sequence have been modelled with 99.5% confidence. This substitution in C terminal of spectrin protein is tolerated since it does not introduce streric clashes.

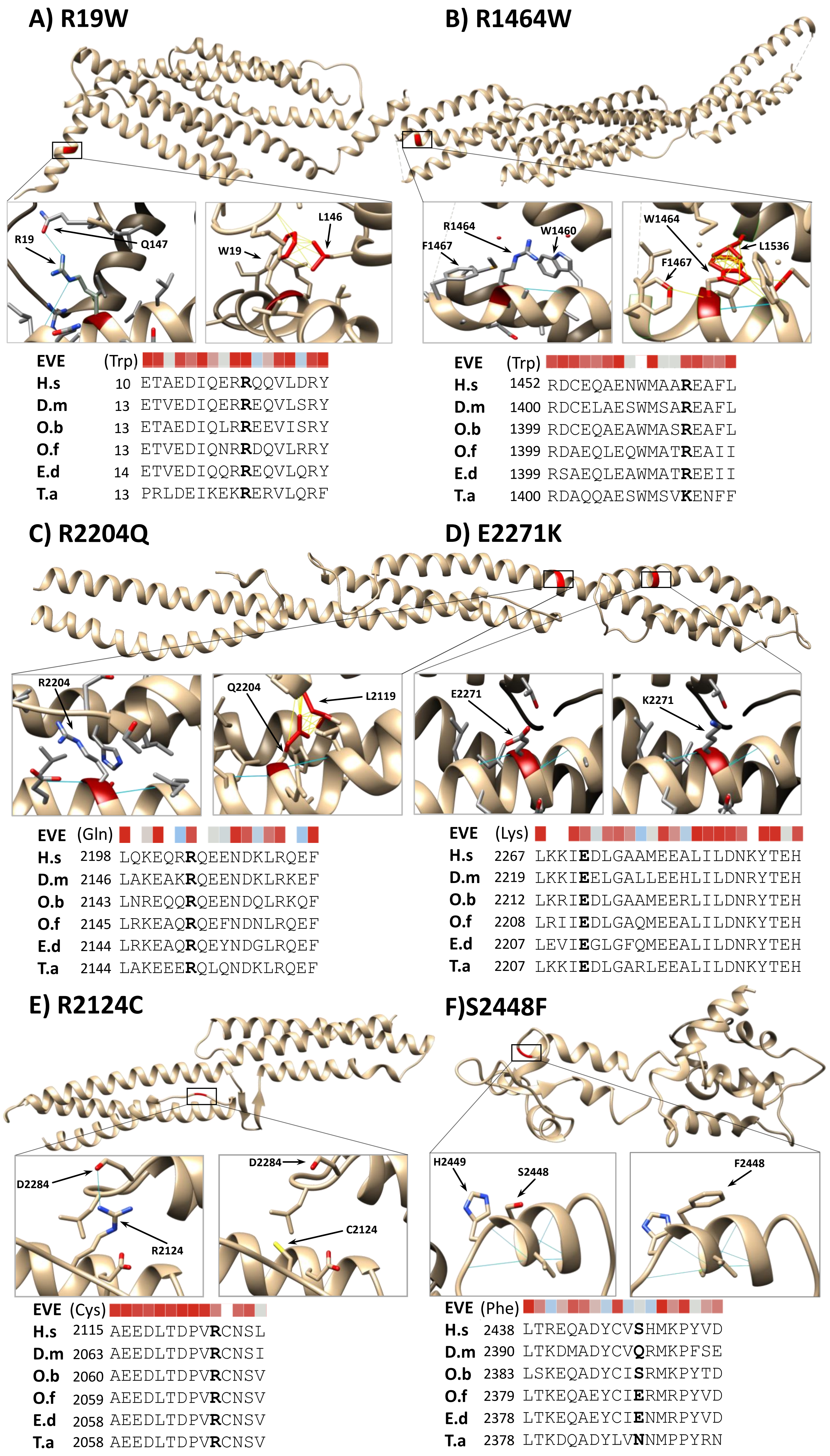
A) R19W
R19
Q147
W19
L146
EVE (Trp)
H.s 10 ETAEDIQERRQQVLDRY
D.m 13 ETVEDIQERREQVLSRY
O.b 13 ETAEDIQLRREEVISRY
O.f 13 ETVEDIQNRRDQVLRRY
E.d 14 ETVEDIQQRREQVLQRY
T.a 13 PRLDEIKEKRERVLQRF
B) R1464W
R1464
F1467
W1460
W1464
L1536
F1467
EVE (Trp)
H.s 1452 RDCEQAENWMAAREAFL
D.m 1400 RDCELAESWMSAREAFL
O.b 1399 RDCEQAEAWMASREAFL
O.f 1399 RDAEQLEQWMATREAII
E.d 1399 RSAEQLEAWMATREEII
T.a 1400 RDAQQAESWMSVKENFF
C) R2204Q
R2204
Q2204
L2119
EVE (Gln)
H.s 2198 LQKEQRRQEENDKLRQEF
D.m 2146 LAKEAKRQEENDKLRKEF
O.b 2143 LNREQQRQEENDQLRKQF
O.f 2145 LRKEAQRQEFNDNLRQEF
E.d 2144 LRKEAQRQEYNDGLRQEF
T.a 2144 LAKEEERQLQNDKLRQEF
D) E2271K
E2271
K2271
EVE (Lys)
H.s 2267 LKKIEDLGAAMEEALILDNKYTEH
D.m 2219 LKKIEELGALLEEHLILDNRYTEH
O.b 2212 LKRIEDLGAAMEERLILDNRYTEH
O.f 2208 LRIIEDLGAQMEEALILDNKYTEH
E.d 2207 LEVIEGLGFQMEEALILDNRYTEH
T.a 2207 LKKIEDLGARLEEALILDNKYTEH
E) R2124C
D2284
R2124
D2284
C2124
EVE (Cys)
H.s 2115 AEEDLTDPVRCNSL
D.m 2063 AEEDLTDPVRCNSI
O.b 2060 AEEDLTDPVRCNSV
O.f 2059 AEEDLTDPVRCNSV
E.d 2058 AEEDLTDPVRCNSV
T.a 2058 AEEDLTDPVRCNSV
F)S2448F
H2449
S2448
F2448
EVE (Phe)
H.s 2438 LTREQADYCVSHMKPYVD
D.m 2390 LTKDMADYCVQRMKPFSE
O.b 2383 LSKEQADYCISRMKPYTD
O.f 2379 LTKEQAEYCIERMRPYVD
E.d 2378 LTKEQAEYCIENMRPYVD
T.a 2378 LTKDQADYLVNNMPPYRN

Supp.Videos:

Video S1: Phenotype of patient 1 with the recurrent *SPTAN1* p. Arg19Trp variant, exhibiting a spastic and scissoring gait.

Video S2: Ataxic phenotype of nine-month-old C57BL/6J mouse with p. Arg1098Gln variant showing obvious motor and coordination difficulty. Mouse model was generated by Miazek et al. [(12)]

References:

1. Davies MD, Leila Elbahy, Tom Fowler, Sue Hill, Tim Hubbard, Luke Jostins, Nick Maltby, Jeanna Mahon-Pearson, Gil McVean, Katrina Nevin-Ridley, Matthew Parker, Vivienne Parry, Augusto Rendon, Laura Riley, Clare Turnbull, Kerrie Woods. The National Genomics Research and Healthcare Knowledgebase. figshare.Dataset 2017.

2. Kircher M, Witten DM, Jain P, O'Roak BJ, Cooper GM, Shendure J. A general framework for estimating the relative pathogenicity of human genetic variants. Nat Genet. 2014;46(3):310-5.

3. Adzhubei IA, Schmidt S, Peshkin L, Ramensky VE, Gerasimova A, Bork P, et al. A method and server for predicting damaging missense mutations. Nat Methods. 2010;7(4):248,9.

4. Vaser R, Adusumalli S, Leng SN, Sikic M, Ng PC. SIFT missense predictions for genomes. Nat Protoc. 2016;11(1):1-9.

5. Richards S, Aziz N, Bale S, Bick D, Das S, Gastier-Foster J, et al. Standards and guidelines for the interpretation of sequence variants: a joint consensus recommendation of the American College of Medical Genetics and Genomics and the Association for Molecular Pathology. Genet Med. 2015;17(5):405-24.

6. Rodrigues CH, Pires DE, Ascher DB. DynaMut: predicting the impact of mutations on protein conformation, flexibility and stability. Nucleic Acids Res. 2018;46(W1):W350-W5.

7. Mehboob S, Song Y, Witek M, Long F, Santarsiero BD, Johnson ME, et al. Crystal structure of the nonerythroid alpha-spectrin tetramerization site reveals differences between erythroid and nonerythroid spectrin tetramer formation. J Biol Chem. 2010;285(19):14572-84.

8. Evans RJ, Davies DR, Bullard JM, Christensen J, Green LS, Guiles JW, et al. Structure of PolC reveals unique DNA binding and fidelity determinants. Proc Natl Acad Sci U S A. 2008;105(52):20695-700.

9. Kelley LA, Mezulis S, Yates CM, Wass MN, Sternberg MJ. The Phyre2 web portal for protein modeling, prediction and analysis. Nat Protoc. 2015;10(6):845-58.

10. Pettersen EF, Goddard TD, Huang CC, Couch GS, Greenblatt DM, Meng EC, et al. UCSF Chimera--a visualization system for exploratory research and analysis. J Comput Chem. 2004;25(13):1605-12.

11. Frazer J, Notin P, Dias M, Gomez A, Min JK, Brock K, et al. Disease variant prediction with deep generative models of evolutionary data. Nature. 2021;599(7883):91-5.

12. Miazek A, Zalas M, Skrzymowska J, Bogin BA, Grzymajlo K, Goszczynski TM, et al. Age-dependent ataxia and neurodegeneration caused by an alphaII spectrin mutation with impaired regulation of its calpain sensitivity. Sci Rep. 2021;11(1):7312.
